# Methodology of Clinical Trials Aimed at Assessing Interventions for Cutaneous Leishmaniasis

**DOI:** 10.1371/journal.pntd.0002130

**Published:** 2013-03-21

**Authors:** Piero Olliaro, Michel Vaillant, Byron Arana, Max Grogl, Farrokh Modabber, Alan Magill, Olivier Lapujade, Pierre Buffet, Jorge Alvar

**Affiliations:** 1 UNICEF/UNDP/World Bank/WHO Special Programme for Research and Training in Tropical Diseases (WHO/TDR), Geneva, Switzerland; 2 Centre for Tropical Medicine and Vaccinology, Nuffield Department of Medicine, University of Oxford, Churchill Hospital, Oxford, United Kingdom; 3 Competence Centre for Methodology and Statistics, Centre de Recherche Public – Santé (CRP-Santé), Luxembourg, Grand Duchy of Luxembourg; 4 Walter Reed Army Institute of Research (WRAIR) Division of Experimental Therapeutics, Silver Spring, Maryland, United States of America; 5 Drugs for Neglected Diseases initiative (DNDi), Geneva, Switzerland; 6 UMR945, University Pierre & Marie Curie, Paris, France & Department of Parasitology, Pitié-Salpêtrière Hospital, APHP, Paris, France; 7 World Health Organization, Neglected Tropical Diseases (WHO/NTD), Geneva, Switzerland; Ege University, Turkey

## Abstract

The current evidence-base for recommendations on the treatment of cutaneous leishmaniasis (CL) is generally weak. Systematic reviews have pointed to a general lack of standardization of methods for the conduct and analysis of clinical trials of CL, compounded with poor overall quality of several trials. For CL, there is a specific need for methodologies which can be applied generally, while allowing the flexibility needed to cover the diverse forms of the disease. This paper intends to provide clinical investigators with guidance for the design, conduct, analysis and report of clinical trials of treatments for CL, including the definition of measurable, reproducible and clinically-meaningful outcomes. Having unified criteria will help strengthen evidence, optimize investments, and enhance the capacity for high-quality trials. The limited resources available for CL have to be concentrated in clinical studies of excellence that meet international quality standards.

## Author Summary

Solid evidence is needed to decide how to treat conditions. In the case of cutaneous leishmaniasis, the diversity of clinical conditions, combined with the heterogeneity and weaknesses of the methodologies used in clinical trials, make it difficult to derive robust conclusions as to which treatments should be used. There also other imperatives - ethical (not exposing patients to treatments that cannot be assessed adequately) and financial (optimize use of limited resources for a neglected condition). This paper is meant to provide clinical investigators with guidance for the design, conduct, analysis and report of clinical trials to assess the efficacy and safety of treatments of this condition.

## Introduction

### Why standardised methodologies for CL

It is important to harmonize and improve clinical trial methodology for cutaneous leishmaniasis (CL); currently, treatment options are few and the quality of the supporting evidence is generally inadequate, making the strength of recommendations for the treatment of this disease inadequate.

To improve on the case management and control of CL, better treatment modalities with reliable evidence of the efficacy, safety, tolerability and effectiveness is required. High-quality clinical trials are essential to determine which therapeutic interventions can confidently be recommended for treating which form of CL. Today, this is unfortunately not the case in numerous instances.

The inadequacies of trials of different treatments of CL has been documented by two WHO-supported Cochrane systematic reviews [1], [2] which included 97 randomized controlled trials on treatments for Old World and American CL. They revealed critical issues related to the methodological quality of the design and reporting of these clinical trials, which make it difficult to compare results, meta-analyse the studies, and draw generalizable conclusions. Weaknesses ranged from the inadequacy of study design (including appropriate controls, endpoints, outcome measures, follow-up times), execution (randomization, allocation concealment, blinding), analyses and reporting (e.g. use of disparate endpoints) [3]. They also found a large number of trials that did not meet basic criteria, and could not be included in the analyses.

This makes a highly compelling and cogent case for defining and harmonizing elements related to the design, conduct, analysis, clinical relevance, and reporting of trials, and ultimately study acquiescence by regulatory agencies. Improving the quality of studies and harmonizing protocols will make meta-analysis more informative and thus strengthen evidence for recommendations on treatment and case management. Furthermore, conducting inadequate trials may lead to inappropriate conclusion, is both unethical and an inefficient use of the limited resources available for research into this neglected disease.

As heterogeneity is an inherent feature of CL (reflecting the variety of species and manifestations), there are obvious challenges in designing and interpreting trials to assess interventions for CL which will allow deriving generalizable results and recommendations.

The objective of this paper is twofold:

- To provide clinical investigators with guidance for the design, conduct, analysis and report of clinical trials of treatments for CL. There is a particular need for standardized methodologies recognizing the complexity of the disease, and for defining measurable, reproducible and clinically-meaningful outcomes.
- To enhance the capacity for high-quality trials. It is clear that the limited resources available for CL have to be concentrated in clinical studies of excellence that fulfil the requirements to conduct good clinical studies and carried out according to international Good Clinical Practice (GCP) standards. It is also clear that disease-endemic countries must be assisted in acquiring the capacities to conduct these trials.

This paper focusses on CL trial-specific issues; it only touches upon more general aspects of clinical trial conduct, which are extensively addressed in a number of relevant papers and documents. For instance the Global Health Trials website [4] offers several resources including a trial protocol tool [5].

### Very diverse disease manifestations and responses to treatment

The collective name of CL comprises several manifestations caused by different *Leishmania* species in the Old and the New World (OWCL and NWCL) and clinical trial methodology should be adapted to this spectrum of conditions. CL is caused by organisms of the *L. mexicana complex* and *Viannia* sub-genus (*L. braziliensis* and *L. guyanensis* complex) in the New World and *L. major*, *L. tropica* and *L. aethiopica* in the Old World. *L. infantum* in both Worlds and *L.* donovani in the Old World can also cause CL. The wide spectrum of clinical manifestations, natural histories and responses to treatment observed in CL patients is accounted for by the combination of parasite's intrinsic differences and patient's genetic diversity.

The time required for natural cure (“self-healing”) is poorly defined and varies widely; it is generally accepted that lesions caused by *L. mexicana* in the New World and *L. major* in the Old World heal spontaneously in a time varying from a few weeks to several months in the majority of patients – except new foci (where the disease tends to be aggressive and self-healing is uncommon), and as opposed to other species (where spontaneous healing barely occurs or requires years). Bacterial super-infections are also frequent and can interfere with healing.

The natural history of the disease must be accounted for when designing a clinical trial. Good knowledge of the disease characteristics at the trial site is essential; it is not possible to extract generalizable data from the published literature. For instance, when considering the placebo arms of randomised controlled trials (RCTs) from the Cochrane systematic review of OWCL^1^, 3-month cure rates for *L. major* were 21% in Saudi Arabia and 53% in Iran with oral placebo. With a topical placebo, they varied from 13% to 63% at 2 months in Iran and were 61% in Tunisia at 2.5 months. For *L. tropica*, cure rates were 0%–10% with oral placebo. In the New World, the information is scarce and more variable, ranging from 0% cure rate at one month in Panama [6] to 37% at 12 months in Colombia [7] for lesions most probably caused by *L. panamensis*. In Guatemala, using topical or oral placebos a 68% cure rate was reported at 3 months for lesions due to *L. mexicana* and only 2% for lesions due to *L. braziliensis* [8], while other studies have reported cure rates of 27% and 39% in the general population at 3 and 12 months respectively [9], [10]. In Ecuador, in a small group of 15 patients, a cure rate of 75% at 1.5 months (no speciation but likely *L. panamensis*) was reported without any treatment [11].

The examples above illustrate the need to acquire and factor in local data on the natural history of disease in order to assess more accurately treatment performance.

A wide variety of treatment modalities has been reported for CL, but none has been shown to be universally effective. Treatment response varies according to a range of factors, including the *Leishmania* species, the patient immune status and age, the number and localization of the lesions, the severity of the disease, the treatment given and the route of administration, etc.

Treatment would benefit both the individual patient but also reduce the burden of human reservoirs in the case of anthroponotic CL, and prevent super-infection and the resulting complications. The choice of treatment, either local or systemic, is usually based on the size, number and localization of lesions, lymphatic spread or dissemination, patient's immune status, cost, risk-benefit and the availability of the treatment itself in the country. Currently available treatment options (systemic and topical) can be found in the WHO 2010 technical report [12].

## Defining trial participants

The characteristics of the participants to be included must be adapted to the specific purpose of each clinical trial and must be representative of the typical patients seen in practice. The relevance of the spectrum composition of the study population to the range of patients seen in practice is of paramount importance especially in phase 3 an 4 trials. The factors that allow or disallow someone to participate in a clinical trial (“inclusion” and “exclusion” criteria, respectively), are used to identify appropriate participants and ensure both their safety and sound conclusions of the study. Establishing common grounds for entry criteria is also important in order to harmonize study populations across trials and facilitate comparability of trials and meta-analyses. It is also important to indicate the encatchment characteristics in terms of area and population, which would help in deciding as to the applicability of the findings of a trial, and ensure that the enrolled patients are representative of the larger patient population in that site (“spectrum composition”).

### Inclusion criteria

**Figure 1:**
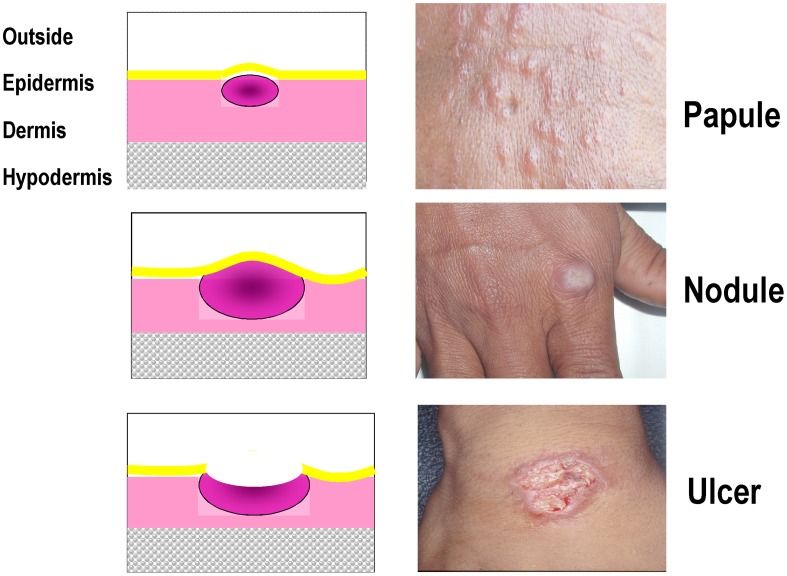
Typical CL lesions.

1. Demography - Define:
  - Gender - are both men and women to be included? CL tends to be gender-sensitive for exposure to infection in certain epidemiological settings (non-domestic and peri-domestic transmission foci), access to treatment and consequences of sequelae. In studies of CL both genders should be eligible for the study. Justify and provide rationale for any reason why either would not be eligible. For some (systemic) treatments, being pregnant or lactating or in child-bearing age may be exclusion criteria. If so required, a proper urine or serum pregnancy test must be documented as negative (generally within 48 hours prior to receipt of the first drug treatment). Repeating pregnancy tests at intervals in the study may be appropriate and should be considered for each study depending e.g. on the drug's residence time in the organism. Females within reproductive age are usually excluded from pre-licensure studies of most investigational new drugs or treatment interventions for safety considerations both for the fetus and the mother. Risks should be carefully weighed against benefits if it is decided to include women in child-bearing age and all necessary measures to prevent exposure during gestation should be set in place – though the risks may not be the same with systemic and topical treatments. Systematically excluding this group may however put them at a disadvantage.
  - Age - all ages or adults only? Provide inclusive age range. In an ideal clinical study any individual with parasitologically confirmed CL would be included. Justify any age groups that would not be eligible for study. The age range will depend on a series of considerations, including the phase of study, the target population in a given area, type of treatment (e.g. invasiveness) and ethical considerations. For example, in early phases of the development of a new drug, most institutional review boards (IRBs) would want to see the drug studied in adults first. The age of the study population must be relevant to the actual population with the disease in a given setting (which, e.g., may be skewed towards young age groups). In addition, data exists showing that age is a determinant of response to treatment [13] possibly based on pre-existing immunity or different drug pharmacokinetics [14], [15]
2. Form of the disease - Define:
  - Which type of cutaneous form? Only localised cutaneous forms are within the scope of this protocol (see exclusion criteria).
  - Morphology of the lesion - refers to the description of lesion appearance using classic dermatologic descriptive terms such as (see also Figure 1; specify which one and whether more than one are accepted):
    - ulcer/ulceration (equivalent terms): meaning that at least part of the lesion is not covered with epidermis - a lesion covered with a crust is considered equivalent to an ulceration (if the crust is removed, the ulceration appears);
    - papule: lesion raised above the skin surface, entirely covered with epidermis, palpable, main diameter smaller than 1 cm;
    - nodule: the enlargement of a papule in three dimensions, solid, easily palpable and greater than 1 cm diameter)
    - plaque: a palpable flat lesion (whether raised above the skin surface or not), greater than 1 cm diameter.
  - Number of lesions - the number of discrete lesions allowed (single or multiple). The reasons for deciding whether all patients with confirmed CL are to be enrolled regardless of the number of lesions present, or only those with a single lesion should be documented and will mainly depend on the type of treatment administered; local treatments (e.g. physical heat treatments or topical creams) may prove either inadvisable (e.g. pain) or impractical to apply to multiple lesions. The number (and location – see below) of lesions vary with the geographic location and depends mostly on the efficiency of the vector; overall, 30–60% of patients will have one lesion, and >80–95% will have <6 lesions. For patients with more than one lesion, it may be possible to select one lesion as the “index lesion” that is used for the diagnosis and evaluation of treatment outcome - when this approach is chosen, one must however consider that a successful treatment is the one that causes all lesions to heal.
    It would be advisable to choose as “index lesion” the uppermost (preferably non-facial), ulcerative, parasite-positive lesion. If two or more lesions are parasitologically positive and are equally uppermost, the subject's left uppermost primary ulcerative lesion will be selected.
  - Size of lesion - either the longest diameter or the surface area of a lesion (Figure 1). The protocol should also provide for whether only the ulcerated area or also the area of surrounding induration is measured. Restrictions for eligibility based on lesion size (e.g. too small to be measured reliably; or too large for topical treatment) should be indicated (provide the justification and rationale). For patients with multiple lesions, it may be possible to express the aggregated size of all lesions. In the future, new handheld devices that easily calculate the volume of the lesion may show advantages over calculating size of lesions using callipers.
  - Topology - the location and distribution of lesions on the body. While in general all patients should be included regardless of the location or distribution of lesions, some situations will warrant exclusion for reasons like: (i) safety - topical treatment of lesion close to the eyes (see exclusion criteria below) or small joints (injections or physical methods); (ii) cosmetic/social stigma - lesions on the face when treatment carries the risk of leaving deep scars. Furthermore, studies have shown that response to treatment may vary based on location on the legs, elbows or knees perhaps due the ease of daily trauma to these sites which may delay healing, or common longer healing of ulcerations on legs [16]. Also, it is well known that lesions on the ears or nose (cartilage) are notoriously slow to heal. If a restriction on the location or distribution of lesions is advisable, provide the justification and rationale.
  - Duration of disease prior to enrolment – the duration of a lesion is an important factor for CL, to account for self-healing. Lesion duration is partially species-dependent; for example, *L. major* tends to heal more quickly, i.e., within approximately within 6–9 months of appearance of the lesion). There is a potential concern that, if one enrolls patients with older lesions, natural healing (notably with *L. major* and *L. mexicana*) may be a confounder.
    The duration is estimated by interrogating the patient. There is no accepted way to standardize this estimate and it is subject to recall bias. Duration of lesion should be included as an inclusion criterion. Depending on the species and the phase of clinical study, only patients with lesions lasting by patient history less than 3 or 6 months would be enrolled.
    For selection of time for inclusion, provide the justification and rationale citing local data to show the natural history of CL in the study focus. While randomisation is expected to even out the distribution between treatment arms, it is possible that this factor is not similar in different arms which could influence healing rates either way, and thus reduce the power of the study, causing the study to fail to detect a difference when there was one. Also there is some concern that early treatment may prevent development of effective immunity, which is an additional reason for collecting information on duration of lesion.
3. Parasitological confirmation of clinically-suspected CL
  - All patients included in a treatment trial for CL must be parasitologically confirmed by a validated technique. Parasitologic confirmation includes visualizing amastigotes by microscopy, visualizing promastigotes in in-vitro culture or by *in vivo* isolation of *Leishmania* from a permissive host, or demonstrating *Leishmania* parasite nucleic acid markers via an appropriately standardized and qualified molecular method. Investigators are encouraged to adopt contemporary molecular technology applied to parasite materials directly from the lesions or the clinical samples without cultivation, to avoid selective amplification of co-existing, non-etiological variants/species.The standard operating procedure (SOP) for each technique used should be included in the study protocol.
    Detection of amastigotes: When done by microscopy, one may consider a quantitative reporting metric such as number of amastigotes per WBCs in oil immersion fields or a semi-quantitative method such as by WHO +,++,+++ method [12]. However, different results may be obtained depending on the sampling, the technique used, the expertise of the microscopist and the extent of examination. In addition, there is no clear evidence that intensity of infection and treatment response are correlated. Numerous studies have shown that the sensitivity of parasitological diagnosis increases from smear to culture to PCR [17]–[20]. Although *Leishmania* parasite speciation (characterization) is usually not required for inclusion in a study, it is highly recommended that characterization be a part of all studies since there is evidence that drugs have different efficacy in different species [10], [13], [21]–[23]. If all parasites cannot be characterised, this can be done on a subset; if no characterisation is done at all, then a strong rationale should be provided as to why not – for instance foci where parasites are known to belong all to the same species. The exact method of characterization must be specified but usually involves protein-based (e.g., isoenzymes) or nucleic acid-based methods (e.g., RFLP-PCR, or PCR followed by sequencing). Enrolling patients on clinical grounds pending the result of parasitologic confirmation should be avoided.
    Serological tests are not sufficient to confirm the diagnosis of CL.
4. Baseline laboratory tests. If required for the subject to have normal renal, hepatic, and haematological functions or other criteria that are judged to be important for their safety especially with respect to specific issues with the interventions being tested. This applies mostly to systemic treatments. The local normal laboratory ranges should be specified. As they are often not known, it would be worth investing in establishing such ranges, at they would be important also for other trials, as well as practice. Common toxicity criteria [24] should be applied to define values outside which subjects are not eligible for enrolment.
5. Informed consent. Subjects must volunteer to participate in the study after being appropriately informed and sign a written informed consent. Participant or parent/guardian able to understand verbal and/or written information [appropriate language at the study site] in which a certified translation of the informed consent is available. There are no unique aspects of informed consent as it applies to a study of treatment interventions in CL. For studies involving children, specific assent may be necessary in addition to the consent obtained from parent/guardian– the age range may vary in different societies and legal requirements.
  Acceptance to participate and be available for the duration of the protocol. Patient needs to be available as protocol requires for the entire duration of supervised treatment and post-treatment follow-up.

### Exclusion criteria

One of more of the following criteria may apply.

1. “Non-localized” leishmaniasis - disseminated or diffuse cutaneous leishmaniasis (DCL), post-kala-azar dermal leishmaniasis (PKDL), mucosal or muco-cutaneous (MCL), leishmaniasis recidivans, or visceral leishmaniasis (VL).
2. Special populations (e.g. pregnant, breastfeeding women, infants, elderly) and serious concomitant illnesses (e.g. known organ dysfunction, or immunocompromised status, including HIV/AIDS) may or may not constitute exclusion criteria depending on the purpose of the trial and the stage of development of the treatment.
3. Lesion close to the nasal, urogenital, and/or anal mucous membranes or to the edge of the lips or eyes are normally excluded when treatment is topical.
4. Consideration should be given to whether palpable lymphnodes in the drainage from a CL lesion warrant or not exclusion for topical treatments. In any case, presence/absence, size, location and quality of lymphnodes should be captured in the initial patient assessment.
5. Known allergy/hypersensitivity to study drugs.
6. Use of prior and concomitant treatments for leishmaniasis, including traditional remedies - e.g. subject must have received no treatment of the current infection during the past e.g., three months; and accepts not to use any other treatment for CL while in the study.
7. Patient conditions requiring the concomitant use of drugs that may interact with the study drug (e.g. QTc prolongation and antimony) or interfere with the course of disease or treatment outcome (e.g. chronic immuno-suppressants, defined as more than 14 days) or other immune-modifying drugs (e.g. for corticosteroids, this will mean prednisone, or equivalent, greater than 0.5 mg/kg/day for more than 5 days).
8. Abusive alcohol ingestion according to CAGE criteria [25]
9. Subject (or their legal guardian) judged by the investigator not capable of understanding and complying with the protocol.

Table 1 illustrates how to apply the different entry criteria based on the type of study (Phase 2–4) and treatment being tested (systemic or topical). The final decision, however, must be taken based on prior knowledge accrued during the pre-clinical and phase I studies.

**Table 1:** Adoption of inclusion- exclusion criteria based on the type of the study and treatment.

	Topical Treatment			Systemic Treatment		
Study Phase	Phase 2	Phase 3	Phase 4	Phase 2	Phase 3	Phase 4
**Criteria**						
Gender	Male & Female	Male & Female	Male & Female	Male & Female	Male & Female	Male & Female
Women of child-bearing age^1^	No	Yes/No	Yes	No	No	Yes/No
Pregnant or breastfeeding^1^	No	No	Yes	No	No	Yes/No
Age	Adults	>5 YO	>2 YO^2^	Adults	>5 YO	All
Type of lesion^3^	Ulcers	All	All	Ulcers	All	All
Number of lesions	1–2	1–5^4^	1–5^4^	1–2	All	All
Size of lesions	≤30 mm^5^	≤30 mm^5^	≤30 mm^5^	≤30 mm	All	All
Localization	Trunk, arms, legs	Trunk, arms, legs, face^6^	Trunk, arms, legs, face^6^	Trunk, arms, legs	All	All
Duration of lesion^7^	≤3 months	≤6 months	≤6 months	≤6 months	≤6 months	≤6 months
Parasitological confirmation	Yes	Yes	Yes	Yes	Yes	Yes
Baseline lab tests, ECG, etc^8^.	Yes/No	No	No	Yes	Yes	No

1 Depending on available pre-clinical and reproductive toxicity data.

2 Age limit due to practical difficulties in measuring skin lesions in very small children.

3 Depending upon the *Leishmania* species and the type of treatment.

4 Due to practical difficulties in treating multiple lesions topically.

5 Due to practical difficulties in treating large lesions topically.

6 Topical treatment of lesions close to mucosae, eyes and ears is generally difficult and/or may pose safety hazard. Decision to include them in advanced phases of clinical evaluation depends on the risks associated with the specific delivery system or formulation used.

7 The decision to set a limit for the lesion age should take into consideration a) the *Leishmania* species –probability that lesions will self-heal within the study time; and b) the difficulty in accurately establishing the age of the lesion from interviewing the patient due to recall bias.

8 Depending on the risk of systemic toxicity based upon pre-clinical toxicity and clinical data available and the route of administration.

## Endpoint - outcome measures and therapeutic assessment

The protocol must identify clearly primary and secondary endpoints for efficacy and safety. The primary efficacy endpoint must be both accurate and robust; the protocol should clarify how and when cure is defined. It is advisable to focus the research on few endpoints that are feasible and attainable within the study, and avoid multiple, diffuse endpoints. Harmonizing efficacy endpoints is essential to allow comparing study results and conducting meta-analyses.

Any procedures applied which may interefere with healing should be standardised upfront and reported in sufficient details. Such would be the case, for standard of care, including dressing, debridement and cleaning of ulcers before and during treatment.

### Efficacy parameters

#### Cure should be defined on clinical parameters

It is generally agreed that cure should be defined based on clinical parameters. Early studies showed that parasitological examination at the end of therapy correlates poorly with the final treatment outcome [26] and relatively few studies have since based their definition of cure on a parasitological outcome. However, it must be pointed out that, for licensure studies, this point may have to be discussed beforehand with regulatory authorities (which will have no particular knowledge of the disease, and apply traditional clinical microbiology criteria).

#### Ulcer surface area should be the primary efficacy endpoint, whenever possible

**Figure 2:**
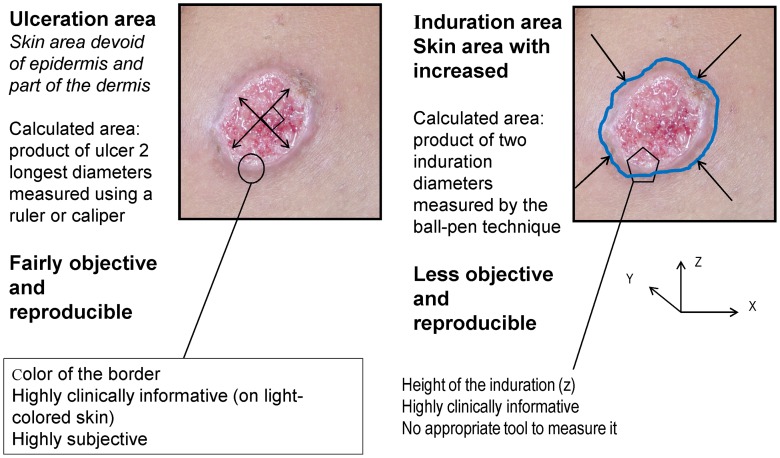
Measuring lesions.

Ideally, a clinically accurate definition would include a combination of five parameters (Figure 2): (i) area of ulceration, when present (x by *y*), (ii) area of induration (*x′* by *y′*), (iii) thickness of induration (z), (iv) colour of infiltrated border, and (v) degree of scaring as a proxy for patient's quality of life.

However, colour and thickness are prone to inter-observer variations and difficult to measure, and quality of life is highly subjective. There is, however, increasing general attention on patient-reported outcomes (PRO) being used as study endpoints. Research into properly constructed PROs should be encouraged. Specifically for CL, this would apply in particular to cosmetic endpoints, like scar assessment methods.

Ulceration area (after debridement and cleaning) is the easiest parameter to measure, and is also clinically meaningful. There is recent evidence that ulceration and induration have parallel evolutions, so both accurately reflect lesion evolution (Buffet, Ben Salah, Grögl et al. Unpublished data).

#### When should induration be used instead of ulcer?

For species causing predominantly nodular lesions (*L. infantum*, *L. mexicana*, *L. aethiopica*), induration area should be used to measure treatment effects. Measuring induration is more difficult than measuring ulcers, and requires training of the study team on e.g. the ball-pen technique, to ensure inter-observer reproducibility. Only areas of “red” or “inflamed” induration should be considered, while hypertrophic scars (where induration no longer reflects an active lesion but rather an aberrant scarring evolution) should be discounted.

Induration should also be used to capture relapses manifesting as purely nodular lesions (i.e., no ulceration). This is a rare situation where parasitological examination should be performed in order to ascribe the new lesion to the parasite. Satellite lesions occur in 5–8% of the CL caused by *L. tropica*. These classical lesions do not always contain abundant parasites and may not require parasitological examination, which is invasive.

#### When should cure be assessed?

The use of single time point at which cure rates are compared between arms is simple and practical, but not fully informative. Time to cure is also important for self-healing CL. Actuarial analysis of multiple sequential observations (e.g. product-limit estimate of time-to-cure using Kaplan-Meier analysis) is also possible though more cumbersome and care must be exercised not to over-estimate clinically non-relevant differences – see section on survival analysis below.

Clinical trials conducted between the late 80's and early 2000's [8], [10], [27]–[29] showed that tissue repair may take several weeks after the causal factor has been removed (i.e., parasites have been killed). Empirically, 6–9 weeks after treatment start is a reasonable compromise – it leaves enough time for most lesions to heal, yet it is not too long for a patient receiving placebo or an ineffective treatment to receive rescue treatment.

In order to both harmonise and simplify procedures, treatment outcome should be assessed on three occasions (counting from the first day of treatment):

1. Day 42–63 (6–9 weeks) for “initial response”, in order to identify early failures. The range is to allow for different healing rates for L. major, L. mexicana (Day 42) and other species (L. tropica, most of the NWCL, Day 63).
2. Day 90±1 week (3 months) for “initial cure”, and
3. Day 180–360±2 weeks (6–12 months) for “definitive cure”, in order to allow for long-term relapses. The overall duration of follow-up will be based on local data on the natural history of disease, as well as practical considerations (e.g. dropout rates with longer follow-up)
4. Visists in-between the above are also encouraged, when feasible.

For a unified, standardised efficacy reporting, a simple, dichotomous outcome definition as either “cure” or “failure” should be adopted, whereby “cure” can only be declared at the end of follow-up (Day 180–360), whereas “failure” can occur at any time (and will require rescue treatment).

**Figure 3:**
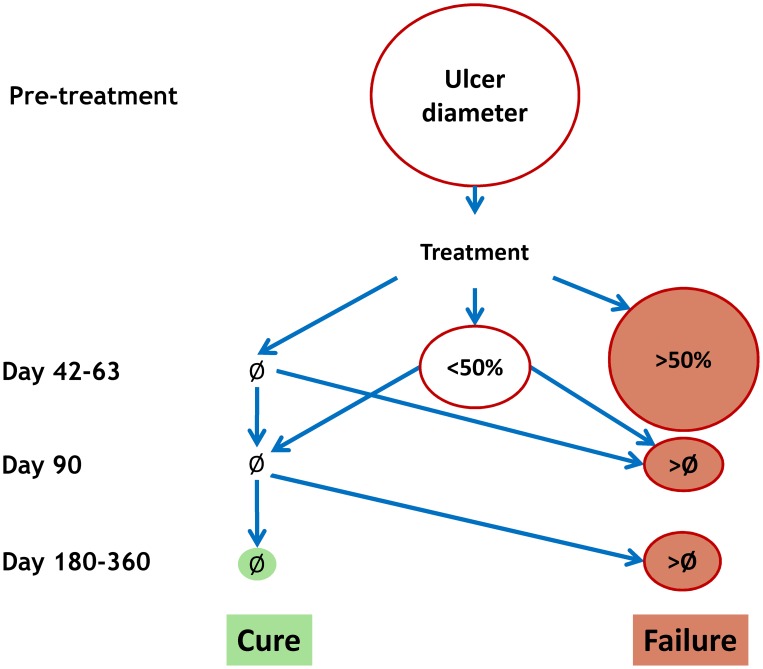
Decision tree for the assessment of treatment outcome. Ø = complete re-epithelialisation; <50% = less than 50% of the initial size; >50% = greater than 50% of the initial size.

Figure 3 describes the decision-tree.

“Cure” is defined as:

- An ulcer that on Day 42–63 is completely re-epithelialised (Ø) and remains so on both Days 90 and 180–360 (no relapse)
- An ulcer that on Day 45 is <50% of the initial size and is completely re-epithelialised (Ø) on both Days 90 and 180–360 (no relapse)

“Failure” is defined as:

- An ulcer that on Day 42–63 is >50% of the initial size
- An ulcer whose size is >Ø at Day 90 or Day 180–360, irrespective of whether it had been re-epithelialised (Ø) before (relapse)

Depending on the natural history of the diseases or its local epidemiological characteristics, additional, secondary parameters may be used to qualify cure, such as the absence of induration, redness or papules around the lesion, or, in case of papules and nodules, parasitological positivity – though after due consideration of its significance.

### Safety parameters

The assessment and reporting of the safety, toxicity and tolerability of treatments, while an essential component of the evaluation, is often overlooked in CL clinical trials. Topical treatments may produce local events at the site of the lesion (like irritation); systemic treatments may cause generalised signs or symptoms, including changes in laboratory values.

Events should be reported and graded using standard nomenclature and criteria of severity. Whenever possible, events must be combined under a syndrome or diagnosis.

It is important to comply with regulations for filing serious events; specific requirements exist for timely reporting accoriding to national regulations (health authorities, regulatory authorities, ethics committees). However, investigators must be alerted to the fact that definitions and rules for reporting may evolve with time and are not fully harmonised between countries.

- Definitions – it is important that clinical trialists understand and use the appropriate definitions - see e.g. relevant documents by the International Conference for Harmonization (ICH; specifically the E6 Guidance on GCP and the E2A guidance on Clinical Safety management [30]), the European Medicines Agency (EMA) [31] and the US Food and Drugs Administration (FDA) [32].
  1. All events, whether considered drug-related or not, should be recorded (adverse event, AE) [33]. An AE is any untoward medical occurrence in a patient or clinical investigation subject administered a pharmaceutical product and which does not necessarily have a causal relationship with this treatment. An AE can therefore be any unfavourable and unintended sign (including an abnormal laboratory finding), symptom, or disease temporally associated with the use of a medicinal (investigational) product, whether or not related to the medicinal (investigational) product.
  2. When a causal relationship with the treatment is established or suspected (“reasonable possibility”) by the investigator, an AE is defined as adverse drug reaction (ADR). In the case of a new medicinal product (aka “investigational new drug”, IND) or its new usages, the ICH guidelines indicate that any noxious and unintended responses should be reported as suspected adverse reaction (SAR) as the drug-event relationship cannot be ruled out. In the case of marketed products an ADR is defined as an event which occurs at doses normally used in man for prophylaxis, diagnosis, or therapy of diseases or for modification of physiological function. It is generally recommended that “ADR” be preferred over “side effect”.
  3. When the AE is “unexpected”, this defines a UAE (Unexpected Adverse Event) – not encountered before (i.e. not in the drug Investigator Brochure for a new product, or not in the summary of product characteristics for a marketed drug) or not at the observed severity, whether it may be anticipated from the pharmacological profile of the drug under investigation or drugs belonging to the same class.
  4. When the AE meets the criteria of being “serious” (which is different from “severe”), this is considered a Serious Adverse Event (SAE). An SAE is any untoward medical occurrence that results in death, is life-threatening, requires inpatient hospitalization or prolongation of existing hospitalization, results in persistent or significant disability/incapacity, or - is a congenital anomaly/birth defect.
- Requirements for reporting. According to the ICH guidelines, the clinical investigator must report an SAE immediately to the sponsor, which in turn must report the event to the relevant authority if the SAE is considered drug-related and unexpected (Serious Unespected Suspected Adverse Reaction, SUSAR). In case the investigator is also the sponsor, s/he has to fulfil all sponsor's responsibilities. Specific local requirements must be taken into account, too.
- When analysing the events, the comparison with baseline (pre-treatment, aka “medical history”) allows defining treatment-emergent AE (TEAE) - defined as any event (sign, symptom, laboratory abnormality) which was either not present prior to the initiation of the treatment or worsened (in either intensity or frequency) with the treatment. Using TEAE helps separating those events that preceed treatment (related to the disease or to the subject's pre-existing conditions) from those that occur or worsen with the treatment. In order to be able to analyse and report on TEAEs, the occurrence and intensity of events must be recorded at baseline (before the treatment is administered), as well as any time post-treatment, and the occurrence and intensity compared.
- Terminology- it would be useful to harmonise the terminology to identify events; while proprietary medical dictionaries exists, the WHO International Classification of Diseases (ICD) is free and can be used for the purpose [34].
- Grading - for grading intensity of events (mild, moderate, severe, very severe), use standardised criteria, e.g. the Common Terminology Criteria for Adverse Events (CTCAE) (version 4.03 [35]) or the Division of Microbiology and Infectious Diseases (DMID), National Institute of Allergy and Infectious Diseases (NIAID), National Institute of Health (NIH) adult and paediatric toxicity tables [36]. It would be useful to consider developing CL-adaped CTCAE criteria.
- Drug-event relationship – it is often difficult to establish and subjective, and depends on previous experience with the use of a treatment. There are therefore differences in the appreciation of events between established and new products, which may introduce biases in unblinded clinical trials. Definitive evidence can only come from de-challenging (the effect disappears) and re-challenging (the effect represents) but this procedure is often difficult to apply.

## Study design

This section treats of study design with a specific focus on issues of special relevance to comparing treatments for CL. In this context, we delve more into types of design (such as non-inferiority trials, adaptive designs) that the typical CL investigator might be less familiar with.

According to the recent WHO treatment recommendations for leishmaniasis, including CL, there are cases (e.g. uncomplicated *L. major*) where an unfavourable risk-benefit ratio (resulting from the combination of a self-curing lesion and the lack of an effective and safe treatment) means that no treatment may currently be recommended (and thus no standard treatment exists to which to compare) [12]. In other cases, cure rates up to or above 90% have been reported following different treatments, though results depend also on the duration of follow-up [1], [3]. However, even when efficacy is high, the risk-benefit of some such treatments is not always well-established, or in favour of the intervention (e.g. systemic toxicity associated with the use of parenteral antimony).

These elements must be accounted for when designing a clinical trial for any specific form of CL. These trials will belong to either of the following types: Phase 2 (safety and dose-finding studies to select the dose and duration of treatment which is safe and effective to be tested further in larger efficacy studies); Phase 3 (randomized controlled trials (RCTs) to establish the value and support the registration of a new intervention with superiority design (over reference treatment or placebo) or non-inferiority design (against a reference standard treatment); or Phase-4 trials (post-registration, when the new treatment is being implemented in the field in conditions that are closer to real life). All studies, whether with or without a direct external comparison, should have at least two arms and be randomized, with few exceptions.

### Randomized comparative designs

#### Comparator (reference) intervention

Current WHO recommendations [12] provide for multiple options, including no treatment, topical or systemic treatment, depending on the species and clinical judgment. Therefore the choice of the reference treatment will have to based largely on local experience and expert opinion – yet supported by reliable data. According to the International Conference for Harmonization (ICH) [37], the choice of a control group should consider its ability to minimize bias, ethical and practical issues associated with its use, usefulness and quality of inference, modifications of study design or combinations with other controls that can resolve ethical, practical, or inferential concerns, and its overall advantages and disadvantages. The guidelines include five types of control groups: i) placebo, ii) no treatment, iii) different dose or regimen of the study treatment, iv) a different active treatment, v) external historical controls (the latter being of very limited use as it carries important biases and raises serious concerns as to between-groups comparability).

Few cases will warrant a placebo or no-treatment arm unless this is as an ‘add-on’ to generally accepted (partially) effective treatment [38]. The choice of giving patients no treatment or a placebo must be on solid scientific and ethical foundations. A no-treatment arm may be justified in case of uncomplicated, self-healing lesions and will provide much needed information on the natural history of disease upon which future studies can be built – although this may be site-specific and non-generalizable. Such an option will however depend on ethical considerations and local regulations.

It is important to be clear as to what is meant by “placebo”; as a placebo should match the active drug, it may be oral or topical – it is difficult to conceive an injectable placebo. Between the two, the only genuine placebo is oral. Basic interventions like cleaning and protecting the lesion against super-infections, as well as topical placebos are known to modify the natural history of the disease, and will likely accelerate the self-healing rate. For clarity, the term “vehicle control” should be preferred over “topical placebo” when it is made of a cream or ointment with only excipients and no active ingredient. This effect on wound healing should be considered in placebo-controlled trials, though the increased cure rate obtained with the intervention over and above “topical placebo” will be difficult to quantify.

#### Superiority design

Whatever the comparator, superiority randomized controlled trials (RCTs) are intended to provide evidence that the test intervention is superior to the control intervention.

Calculations and examples follow. The basic statistical elements to be considered in designing a trial are summarized in Box 1.

**Box 1. Statistics – Definitions**

- α (probability of a type I error) is the probability of erroneously rejecting the null hypothesis (i.e. recommending a medicine with no advantages) given that the null hypothesis is true;
- β (probability of a type II error) is the probability of erroneously failing to reject the null hypothesis (i.e. keeping a good medicine away from patients) given that the alternative hypothesis is true
- 1- β (power) quantifies the ability of the study to find true differences of various values of δ (see below). It expresses the chance of correctly identifying the alternative hypothesis, and to correctly identifying a better medicine.
- Δ is the minimum difference between groups that is judged to be clinically important - i.e. the minimal effect which has clinical relevance in case management.

The choice of the values of type one error rate, α, and power, 1- β (i.e. how stringent the study will be), as well as the expected cure rates with the control and the improvement to be detected for the test intervention will determine the sample size of the study. Noteworthy, reliable efficacy data for the comparator arm are needed; wrongly estimating the efficacy of the comparator treatment may result in the study being underpowered, hence failing to produce the intended results.

When the number of arms is >2 (i.e. >1 test intervention or dose), this will have to be accounted for in sample size calculation and result in a larger sample size per group, other things being equal, in order to allow for multiple comparisons.

The study may be designed to compare proportions (cure rates) between the control and test intervention, but also means (e.g. of size of lesions). A non-significant result (i.e. no significant difference detected) does not imply that the two treatments are equal [39].

**Figure 4:**
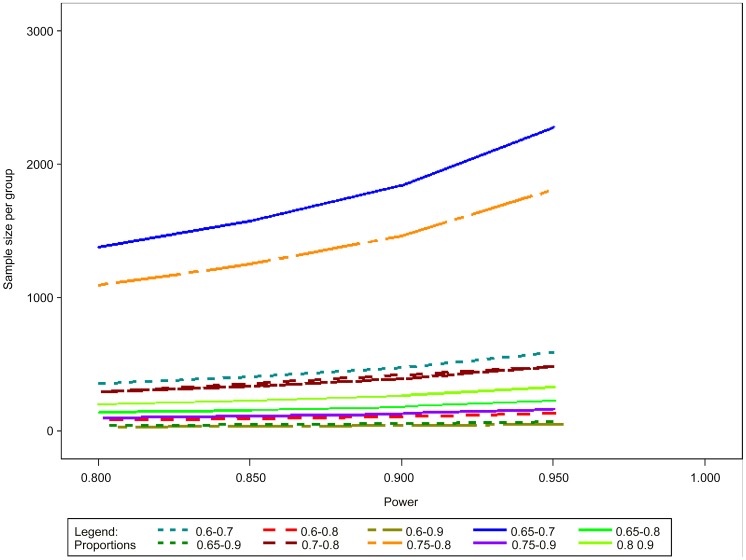
Sample size calculations for comparative superiority trials.

Examples of assumptions and their implications in terms of sample size calculations are provided in Figure 4 and Table 2, assuming: a two-tailed test, α = 0.05; power (1-ß) = 0.80, 0.85 or 0.90; success rate of the comparator drug = 60–90%; and δ = 10–30%. The larger the δ, and the more effective the reference intervention, the smaller the sample size. In the typical example of a superiority design with the reference treatment being 80% effective, expecting a 10% difference with the test treatment (90% effective) with power = 0.80, 199 patients per arm would need to be recruited. For comparison, a 10% difference with a reference treatment that is 70% effective will require 294 patients.

**Table 2:** Sample size calculations for superiority trials.

Control treatment Success Rate	Test treatment Success Rate	Power	N Per Group
0.6	0.7	0.80	356
0.6	0.7	0.85	407
0.6	0.7	0.90	477
0.6	0.7	0.95	589
0.6	0.8	0.80	82
0.6	0.8	0.85	93
0.6	0.8	0.90	109
0.6	0.8	0.95	134
0.6	0.9	0.80	32
0.6	0.9	0.85	36
0.6	0.9	0.90	42
0.6	0.9	0.95	52
0.65	0.7	0.80	1377
0.65	0.7	0.85	1575
0.65	0.7	0.90	1842
0.65	0.7	0.95	2278
0.65	0.8	0.80	138
0.65	0.8	0.85	158
0.65	0.8	0.90	185
0.65	0.8	0.95	228
0.65	0.9	0.80	43
0.65	0.9	0.85	49
0.65	0.9	0.90	57
0.65	0.9	0.95	70
0.7	0.8	0.80	294
0.7	0.8	0.85	336
0.7	0.8	0.90	392
0.7	0.8	0.95	485
0.75	0.8	0.80	1094
0.75	0.8	0.85	1251
0.75	0.8	0.90	1464
0.75	0.8	0.95	1810
0.75	0.9	0.80	100
0.75	0.9	0.85	114
0.75	0.9	0.90	133
0.75	0.9	0.95	164
0.8	0.9	0.80	199
0.8	0.9	0.85	228
0.8	0.9	0.90	266

Efficacy in the reference arm from 60–80%, delta 10–30%, alpha error 0.05, power 80–95%.

In addition, in calculating the sample size, allowance should be made for losses to follow-up - a parameter which is very much site-specific.

The intent-to-treat (ITT) is generally considered the choice population for analysis; it comprises all patients randomized who gave informed consent and received any amount of the assigned intervention at least once. The practical problem in applying ITT is that it requires measurement on all patients whether or not they are still adhering to the protocol. Thus as soon as one has ‘loss to follow-up’ it is not possible to apply a pure ITT analysis. This population reflects treatment effects in conditions that are closer to those encountered in routine use, as opposed to the per-protocol (PP) population, which is restricted to the patients without major protocol deviations who are evaluable at the planned visit for efficacy assessment and thus measures the pure treatment effect (“evaluable patients' analysis”). The mITT population definition is used to overcome the bias of the ITT population. It is a subset of the ITT population allowing for the exclusion of patients due to non compliance or missing outcome. Conclusions will be drawn from the results on the primary criteria calculated on the ITT or the modified-ITT (mITT) population.

#### Non-inferiority design

Non-inferiority trials are intended to show that the new intervention is no worse than the standard drug by some margin Δ (the non-inferiority margin), defined as the largest clinically acceptable difference [40]; it should be smaller than differences observed in superiority trials of active comparator [41].

The non-inferiority design has become increasingly popular in malaria and tuberculosis (where very effective treatments exist), but is rarely used in leishmaniasis; so far, it has been used for visceral leishmaniasis (VL) randomized controlled trials in India [42], [43] and East Africa (DNDi clinical trials.gov NCT01067443).

The choice of the non-inferiority margin is very important as it governs the validity of the trial, and has also ethical implications [44]. The objective is to avoid harmful treatment to be declared non-inferior, and to retain a treatment that brings a true benefit for the patient [41]. The decision should be based on previous studies with the reference treatment and the minimally important effect that one wants to observe with the new treatment which would provide additional benefit for the patients.

In order to identify the correct Δ, it has also been proposed to compare (i) the two-sided 95% confidence interval of the difference between the test and the reference treatment to (ii) a two-sided 95% CI of the difference between the reference treatment and the placebo based on historical data and meta analyses (if such data are available) [45]. Virtual comparison methods are also available, whereby the new treatment is compared to a putative placebo by synthesizing the estimated effectiveness of the former versus an active control and the estimated effect of the latter versus the placebo [46].

It is important to note that defining the Δ is not a mere statistical exercise; it requires consideration of what is a clinically acceptable failure rate, in the context of other factors, such as practicalities (duration of treatment, route of administration) and costs.

Calculations and examples follow. The basic elements to be considered in designing a non-inferiority trial are similar to those of a superiority design. The difference is in the choice of the margin and the test used to compare the treatment estimates. When success or failure rates are used to measure treatment effects, it is common to compare the 95%CI lower limit to the non-inferiority margin. However, in the case of proportions, it should be also of interest to compare risk ratios (RR) or odds ratios (OR) with a non-inferiority margin specified on the RR or OR scale.

In the examples that follow we work with proportions and 95%CI. The sample size is calculated based on the expected proportion of events in the reference arm (80%, 85%, 90% or 95%), the expected true difference in proportions between the reference and the tested treatment arms (0%), α risk = 0.01, unilateral hypothesis, and power (1-ß) = 90% the equivalence margin defined as acceptable for concluding that a tested treatment is not inferior to the reference arm (from 5% to 10%; meaning that one is prepared to accept that the test treatment is 5% or 10% less effective than the reference treatment).

The larger the Δ, and the more effective the reference intervention, the smaller the sample size. Using a reference treatment that is 80% effective, the sample size varies from 1667 (5% Δ) to 417 (10% Δ); similarly, for the same Δ = 10%, the sample will be 124 when the reference treatment is 95% effective.

**Figure 5:**
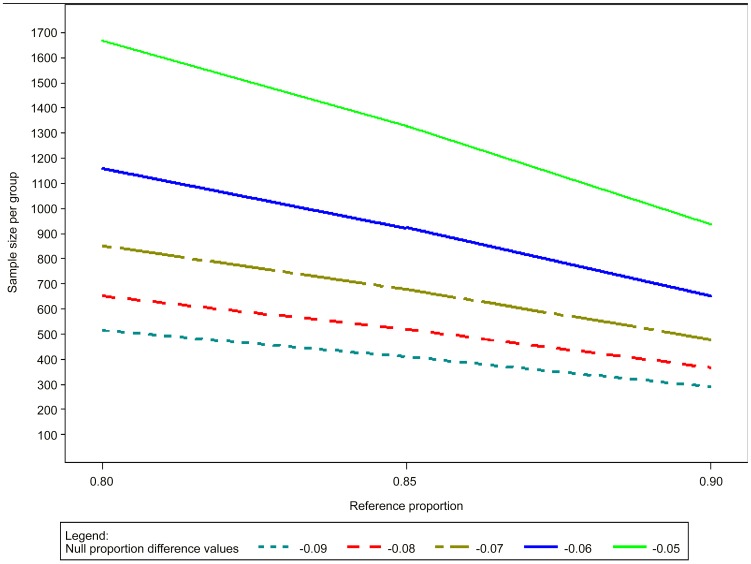
Sample size calculations for comparative non-inferiority trials.

The total sample size would allow an assumption on the expected proportion of drop-outs (5% for instance) and multiply by 2 (groups). In case of more than 2 groups being studied, the calculation will have to allow for an adjustment for multiplicity such as the so-called Bonferoni correction. More results are presented in Figure 5 and Table 3.

**Table 3:** Sample size calculations for non-inferiority trials.

Non-inferiority margin	Reference treatment Success Rate	N Per Group
−0.05	0.80	1667
−0.05	0.85	1328
−0.05	0.90	938
−0.05	0.95	495
−0.06	0.80	1158
−0.06	0.85	923
−0.06	0.90	651
−0.06	0.95	344
−0.07	0.80	851
−0.07	0.85	678
−0.07	0.90	479
−0.07	0.95	253
−0.08	0.80	651
−0.08	0.85	519
−0.08	0.90	367
−0.08	0.95	194
−0.09	0.80	515
−0.09	0.85	410
−0.09	0.90	290
−0.09	0.95	153
−0.1	0.80	417
−0.1	0.85	332
−0.1	0.90	235
−0.1	0.95	124

Efficacy in the reference arm from 80%–95%, delta 5–10%, alpha error 0.01, power 90%.

The Non-inferiority margin represents the smallest acceptable difference with respect to the success rate with the reference treatment.

These calculations show the importance of the non-inferiority margin and the proportions for the reference treatment. When the α risk and power are fixed, the sample size can grow exponentially whenever a little change is done in the assumptions.

Between the ITT and the PP populations, ITT may bias the results toward equivalence, which could make a truly inferior treatment appear non-inferior [41], [47]–[49]. ITT analysis carries the risk of falsely claiming non-inferiority [50] although this may not always be the case [51] (reviewed and discussed in Piaggio et al [52]).

According to Abraha et al [47], in non-inferiority trials “excluding participants who did not adhere fully to the protocol can be justified. Exclusions may, however, affect the balance between the randomized groups and lead to bias if rates and reasons for exclusion differ between groups [53], [54]”. The current thinking of regulatory agencies is that the study objective should be achieved in both the ITT and PP populations, especially in a non-inferiority trial [40]. However, Maltilde-Sanchez et al [55] argue that this “does not necessarily guarantee the validity of a non-inferiority conclusion and a sufficiently powered PP analysis is not necessarily powered for ITT analysis”. These authors propose to perform a new maximum likelihood-based ITT analysis arguing that it could address “the potential types and rates of protocol deviation and missingness that might occur in a non-inferiority trial” and that “prior knowledge regarding the treatment trajectory of the test treatment versus the active control at the design stage” should be collected “so that a proper analysis plan and appropriate power estimation can be carried out”.

Illustrating the divergent conclusions toward non-inferiority between the ITT and PP populations is outside the scope of this work. Neverthless the examples provided in Table 4 (which use rates derived from published NWCL studies at 6–12 months of follow-up) illustrate how much exclusions can influence the sample size required to prove non-inferiority: the more patients are excluded and the less effective the reference treatment is, the larger the sample size required for a given non-inferiority margin – obviously the sample size decreases when the non inferiority margin increases. This means that different conclusions as to non-inferiority may be reached on the ITT vs. the PP populations. Therefore, special attention must be paid to minimizing losses to follow-up and numbers of patients deemed non-assessable, both of whom would be deducted from the PP population.

**Table 4:** Samples size calculation (N per group) for non-inferiority trials.

		reference treatment success rate						
delta	exclusions	60%	65%	70%	75%	80%	85%	90%
6%	0%	1736	1646	1519	1356	1158	923	651
	5%	1823	1728	1595	1424	1216	969	684
	10%	1910	1811	1671	1492	1274	1015	716
	15%	1996	1893	1747	1559	1332	1061	749
	20%	2083	1975	1823	1627	1390	1108	781
	25%	2170	2058	1899	1695	1448	1154	814
8%	0%	977	926	855	763	651	519	367
	5%	1026	972	898	801	684	545	385
	10%	1075	1019	941	839	716	571	404
	15%	1124	1065	983	877	749	597	422
	20%	1172	1111	1026	916	781	623	440
	25%	1221	1158	1069	954	814	649	459
10%	0%	625	593	547	489	417	332	235
	5%	656	623	574	513	438	349	247
	10%	688	652	602	538	459	365	259
	15%	719	682	629	562	480	382	270
	20%	750	712	656	587	500	398	282
	25%	781	741	684	611	521	415	294

Success rate ranging 60–90%; exclusions ranging 0–25%; non-inferiority margin (delta) 6%, 8% and 10%.

### Other randomized designs

#### Precision estimate

A precision estimate can be used when one can estimate success/failure rates or means as well as mean difference from previous studies done in a different environment or time period. The objective is therefore to evaluate this estimate and its variability in a new population.

Examples of sample size with precision estimate [56] if the required success rate is
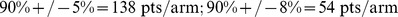


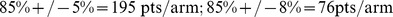



The precision estimate is used in the case of non-comparative design, therefore it cannot judge the efficacy of a treatment comparatively to placebo or an active treatment. It could be used however for dose-finding.

#### Adaptive designs

These designs are meant to allow choices amongst various drugs and regimens (dose, duration) systematically, as quickly and effectively and with as few patients as possible. The term includes group sequential designs, sequential methods and methods to stop earlier trials with superiority or non-inferiority designs.

Adaptive trials designs are increasingly used to improve efficiencies in the R&D process. This approach allows redesigning the trial based on the information acquired through interim analyses, which may result in changing the sample size, the number of arms, or other elements. Sequential and group sequential trials are a special case of adaptive trials where several interim analyses are done in order to complete earlier the trial based on the accumulated information. We will concentrate here on sequential methods, and more specifically on the Whitehead triangular test, a graphical methods defining with boundaries which allows for early rejection or non-rejection of H0.

Examples using the Whitehead triangular test [57] follow. In this example, the hypothesis to be tested will be a difference of 8% between the failure rate (in %) of each group and the boundaries calculated for 10 discrete stages of evaluation.

The type I and type II risk are commonly set at α = 0.05, power (1-ß) = 0.80 i.e. the risk to reject an effective treatment is 5% and the chance for the study to find an effective treatment is 80%. The null proportion is set at 0.1 and the alternate proportion is set at 0.18, 0.20 and 0.25. These assumptions mean that if the failure rate <10%, efficacy is considered adequate, and if the failure rate ≥25% efficacy is insufficient. In terms of probabilities, it can be written that the boundaries of the test are calculated for H0(p≥p0) and Ha(p<pa) with p0 = 0.25 and pa = 0.10.

**Figure 6:**
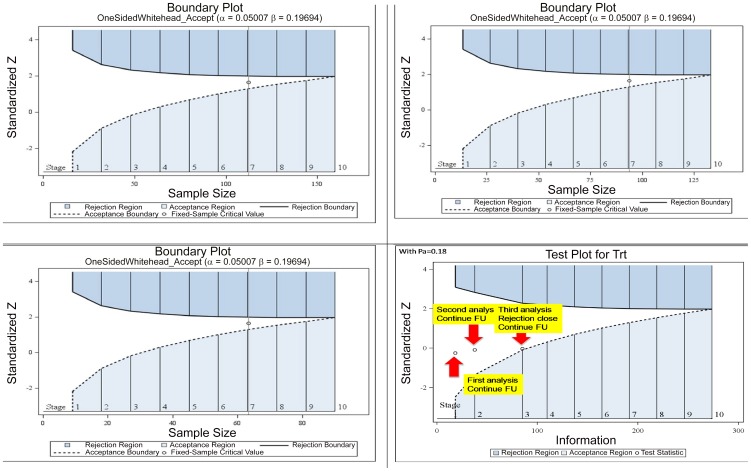
Boundaries of the one-sided triangular test. Left to right; top to bottom: pa = 0.18, pa = 0.20, pa = 0.25, example of sequential analyses with modeled data.

Different sample sizes estimated when varying pa, the alternate proportion of failure: pa = 0.18: min = 8, max = 80; pa = 0.20: min = 7, max = 67; pa = 0.25: min = 5, max = 46 see Figure 6, Table 5, provides an example of calculations for a two-sided test.

**Table 5:** Sample size calculation for the one-sided triangular test.

	one-sided						two-sided	
	Pa = 0.18		Pa = 0.20		Pa = 0.25		Pa = 0.20	
Stage	N per group	Cumulated information Z statistic	N per group	Cumulated information Z statistic	N per group	Cumulated information Z statistic	N per group	Cumulated information Z statistic
1	8	27	7	23	5	16	8	27
2	16	55	14	47	10	31	16	53
3	24	82	20	67	14	44	24	80
4	32	110	27	90	19	60	32	107
5	40	137	34	113	23	72	40	133
6	48	165	40	133	28	88	48	160
7	56	192	47	157	32	101	56	187
8	64	220	54	180	37	117	64	213
9	72	247	60	200	41	129	72	240
10	80	274	67	223	46	145	79	263

pa = 0.18, 0.20 and 0.25; and two-sided test, pa = 0.20.

The triangular test is not without shortcomings, especially in the context of diseases like CL: (i) it is most effective when early end-points exist, which is not the case for CL, though one could consider use of a surrogate marker e.g. 50% re-epithelisation at 42–63 days or another clinically-relevant parameter; (ii) it also requires an efficient (on-line) data-management system in place and a constant interface with a statistician.

The advantages of sequential methods such as the triangular test is that they allow the analysis of the cumulated information at each step, early stopping (when treatment proves effective (p0) or ineffective (pa)), non-comparative and comparative designs, and can eventually result in shortening study duration and reducing the number of subjects to be exposed. As with the fixed sample designs, several treatments (or doses) can be tested in parallel, which is particularly useful for dose-finding (Phase 2) studies.

It would also be possible to conceive a design combining sequentially in a single study (1) screening of potential treatments (one-sided triangular test applied to multiple non-comparative studies as required) and (2) comparing the so selected treatment to the reference treatment (two-sided triangular test).

#### Survival analysis

In a trial testing a new drug, one has to make assumptions on the number of subjects who will not complete the study for any reason. These subjects may not be properly accounted for in the typical ITT or PP populations analysis because they would not have reached an endpoint that makes them qualify for the analysis - in the first case they will be counted conservatively as failures (though they are not demonstrated failures) and in the latter they will be discounted. When dealing with cure rates, one way to circumvent this problem is to use survival (time-to-event) analysis whereby the information accumulated by a subject while on study is accounted for up until the time that s/he drops out of the study or reaches a study endpoint. Withdrawals such as drop-outs, failures or deviations will be censored at the time such event occurs and accounted for, for as long as the subject has been on study.

While this approach is rarely used in CL [58], [59], it would have also the additional advantage of accounting for time-to-healing, which is an important consideration when comparing treatments, or comparing treatment to a placebo (because of variable tendency to natural healing and effects of (topical) placebos on the natural rates of recovery).

Specifically, interventions would be assessed based on the survival estimate of healing at a specific day (e.g. end of follow-up) evaluated for instance using the Kaplan-Meier [60] method (other methods exist) as shown in

**Figure 7:**
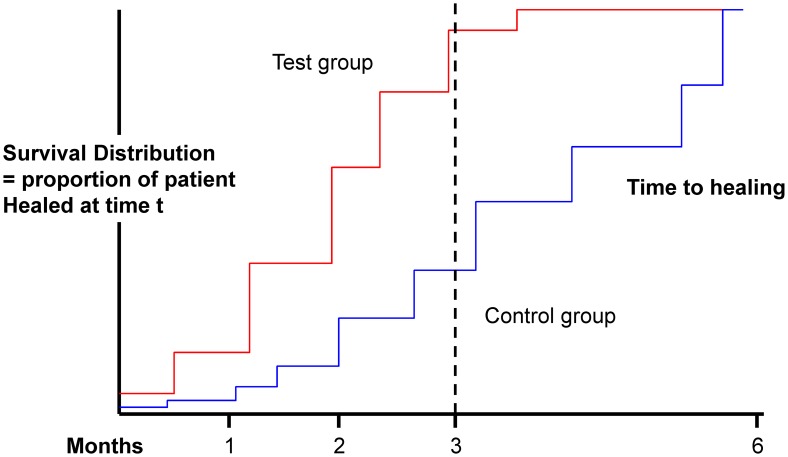
Kaplan-Meier analysis (product-limit estimate of time to event).

Figure 7. It is advisable to include denominators at each time-point of the plot to show the decreasing numbers of patients contributing to the analysis as time goes on.

Outcomes between arms are normally compared using the Log-Rank test, or the proportional hazard model (which allows adjustment for independent factors; furthermore, it estimates also the relative risk (hazard ratio) with one arm over the other one).

Survival analyses can be applied both to superiority and non-inferiority trials, but sample size calculation should be adapted in the latter case (Vaillant & Olliaro, manuscript in preparation).

Herewith we provide an example of a sample size calculation for a non-inferiority trial based on the assumption of a 3-months study duration and a cumulative drop-out rate of 10%. With a type one error α = 1% and a power 1-β = 90%, assuming a cure rate of 80% with the reference treatment and non-inferiority margins of 10%, 7% or 5%, the total sample size required to demonstrate non-inferiority would be 1030, 2020 or 3842 patients respectively. Additional calculations with cure rates of 85% and 90% are also presented in Table 6

**Table 6:** Sample size calculation for the comparison of two survival curves.

Alpha	Power	Test treatment Success rate	Comparator treatment Success rate	Delta	Total sample size
0.01	0.9	80%	70%	10%	1030
0.01	0.9	80%	73%	7%	2020
0.01	0.9	80%	75%	5%	3842
0.01	0.9	85%	75%	10%	884
0.01	0.9	85%	78%	7%	1700
0.01	0.9	85%	80%	5%	3190
0.01	0.9	90%	80%	10%	706
0.01	0.9	90%	83%	7%	1320
0.01	0.9	90%	85%	5%	2422

the log-rank test in a non-inferiority design.

**Figure 8:**
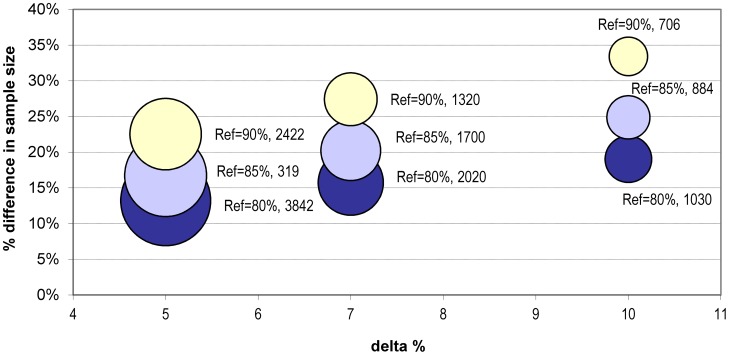
Differences in sample size for a non-inferiority trial when calculated using rates or allowing for survival analysis. Sample size expressed as % underestimation when calculated using rates vs. survival analysis; delta set at 5, 7, 10%; efficacy of comparator arm (Ref) set at 80% dark blue; 85% pale blue; 90% pale yellow. The size of the bubble is proportional to the sample size (figure next to the bubble).

When comparing calculations made allowing or not for product-limit estimate analysis, the latter appear to underestimate systematically the sample size by a factor that is proportionally higher as the δ and reference treatment efficacy increase (from 13% with δ = 5% and 80% efficacy to 33% with 10% δ and 90% efficacy (Figure 8)) and the total sample size decreases (from 3842 to 706 patients and 3334 to 470, respectively).

#### Concluding remarks on the choice of the appropriate design for CL trials

This section provides general directions as to the choice of the appropriate trial design for CL. Against the backdrop of the general lack of standardization and inadequate design [3] in CL clinical trials, as well as the considerations listed above, different designs will befit different questions:

- In early phases (e.g. Phase 2 studies), whereby doses, durations (and permutations thereof), or drug combinations are investigated, it is important to minimize the time and number of subjects needed to reach a conclusion. This is important both for efficiency and ethical reasons. In this case, a precision estimate or a sequential study design may be useful; the latter would allow flexible designs including combinations of non-comparative and comparative selections.
  Additional work should be conducted to identify and validate early markers of treatment outcome, and to understand better the natural history of the different forms of CL. Methods should also be sought to factor self-healing rates into the assessment of benefits from treatment.
- When comparing a new intervention (such as one identified through the process above) to an existing one, the choice between a superiority and a non-inferiority design will depend on the efficacy levels of the available treatment. If the efficacy is considered not enough, the superiority design is required; one will have to decide how much better the new treatment will have to be over and above the old one in order to be preferred and warrant use. If the existing medication is considered effective but with shortcomings that make it, for instance, difficult to apply, or unsafe, or expensive or else, then the non-inferiority design will be appropriate; in this case one would be prepared to accept a trade-off between the new treatment being potentially less effective (and will have to decide by how much) and other benefits (e.g. easier to use, safer, less expensive, or else). Noteworthy, where a non-inferiority design was planned but results are sufficiently impressive, it remains possible to declare superiority with no multiplicity problem since the switching of non-inferiority to superiority requires a simple closed test procedure [61], [62].

## Study registration and reporting

**Figure 9:**
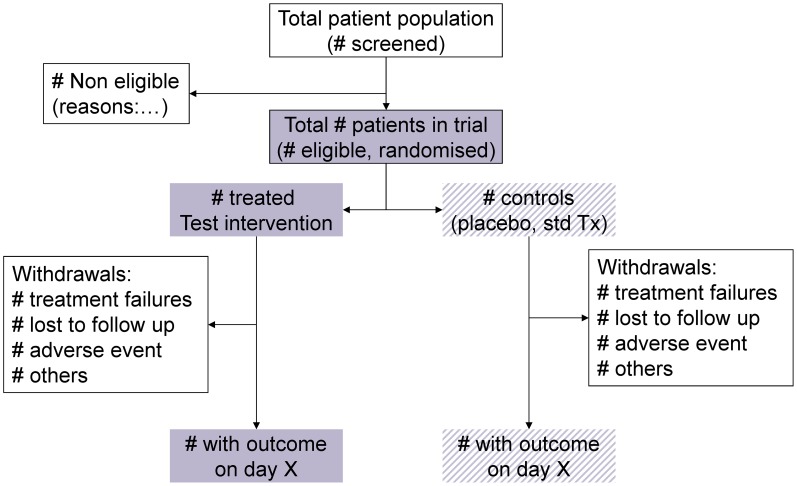
Study flow diagram and patient attrition according to the CONSORT statement.

All trials should be registered (see: the WHO International Clinical Trials Registration Platform (WHO-ICTRP) and reported, whether the results are favourable, unfavourable or inconclusive – both for ethical and scientific reasons. Traditionally, the importance of negative results has been underestimated both by researchers and publishers; publishing only positive results will bias knowledge. The CONSORT checklist (study design, analysis and interpretation) and flow diagram (patient attrition throughout the study) should be followed [63]. All major journals today do not publish papers on trials that have not been registered and do not follow the CONSORT guidelines (see example in Figure 9).

The protocol must be clear as to the population for analysis – typically: intent-to-treat (ITT), modified ITT (mITT) and per-protocol (PP). The basis for exclusion of patients from the analysis must be provided. Patients withdrawn because they could not tolerate treatment or because they required rescue treatment must be accounted for. The analytical plan should be finalised before freezing the data for analysis.

Like any other trial, an appropriate data management process is critical in order to have high-quality data, statistical analyses and results. For this purpose, the data management software adopted must provide a secure location for the clinical data, user rights and profiles along with password protection, as well as an audit trail. Capacity for data management is often scarce in CL-endemic countries, including both the availability of appropriate software with auditable track, and trained data managers. In these countries there is also a general shortage of statisticians to help design and to analyse and report on trials. Capacity building efforts should be organized to increase competences of research teams in this important area.

## Complying with regulations

Clinical trials must be conducted in accordance with current international standards of Good Clinical Practice (GCP), an international ethical and scientific quality standard for designing, conducting, recording and reporting trials that involve the participation of human subjects. Compliance with this standard provides public assurance that the rights, safety and well-being of trial subjects are protected, consistent with the principles that have their origin in the Declaration of Helsinki, and that the clinical trial data are credible. When GCP standards are followed, the quality of data from clinical trials is adequate to make informed clinical and policy decisions.

There is a belief among some that GCP guidelines are only for “registration” studies and not for all clinical trials. However, the principles of GCP should be applied to all clinical studies with any intervention conducted at any stage of development that may have an impact on the safety and well-being of human subjects. Implementation of GCP procedures requires initial training and practice and is best served when trial personnel at a site accept and understand a culture of GCP. Maintaining a GCP environment requires constant training and reinforcement and is a process that requires continuous growth in a site and personnel. Accepted GCP standards include those published by the International Conference on Harmonization (ICH) and the World Health Organization (WHO). The ICH GCP guideline is published under Efficacy (E6) and is often referred to as ICH E6 GCP guideline [64]. A summary review of the principles of GCP are found in the WHO handbook [65].

At the same time, it should be clear that GCP is not about dogma, but rather patient's care and reliability of data, and that the context within which trials occur should be accounted for. A proper balance between the goals of the clinical study and the documentation required has been proposed [66]. The amount of written documentation and the degree of detail required by GCP procedures can be a shock to investigators not used to working in this environment. Although the conduct of clinical trials under GCP with external monitors and proper data management will inevitably increase the cost of studies, it is imperative that higher quality studies in CL be conducted.

For all trials involving human subjects, ethics review and approval must be sought from appropriate boards/committees at the institution (local and/or international) and/or country level as required. It is imperative that all clinical studies are conducted in accordance to the international and country regulations and laws.

## Disclaimer

The opinions expressed in this paper are those of the authors; the authors alone are responsible for the views expressed in this publication and they do not necessarily represent the decisions, policy or views of the WHO.

Material has been reviewed by the Walter Reed Army Institute of Research. There is no objection to its presentation and/or publication. The opinions or assertions contained herein are the private views of the author, and are not to be construed as official, or as reflecting true views of the Department of the Army or the Department of Defense.
